# Rapid and Sensitive Lateral Flow Immunoassay Method for Procalcitonin (PCT) Based on Time-Resolved Immunochromatography

**DOI:** 10.3390/s17030480

**Published:** 2017-02-28

**Authors:** Xiang-Yang Shao, Cong-Rong Wang, Chun-Mei Xie, Xian-Guo Wang, Rong-Liang Liang, Wei-Wen Xu

**Affiliations:** 1 Institute of Antibody Engineering, State Key Laboratory of Organ Failure Research, School of Laboratory Medicine and Biotechnology, Southern Medical University, Guangzhou 510515, China; s1002297748@126.com (X.-Y.S.); plum225@126.com (C.-M.X.); liang.rongliang@163.com (R.-L.L.); 2 Department of Laboratory Medicine, Nanfang Hospital, Southern Medical University, Guangzhou 510515, China; cynthiawang21@hotmail.com; 3 R&D Center, DaRui Biotechnology Co., Ltd., Guangzhou 510655, China; 13250293361@163.com

**Keywords:** time-resolved, immunochromatography, procalcitonin (PCT)

## Abstract

Procalcitonin (PCT) is a current, frequently-used marker for severe bacterial infection. The aim of this study was to develop a cost-effective detection kit for rapid quantitative and on-site detection of PCT. To develop the new PCT quantitative detecting kit, a double-antibody sandwich immunofluorescent assay was employed based on time-resolved immunofluorescent assay (TRFIA) combined with lateral flow immunoassay (LFIA). The performance of the new developed kit was evaluated in the aspects of linearity, precision, accuracy, and specificity. Two-hundred thirty-four serum samples were enrolled to carry out the comparison test. The new PCT quantitative detecting kit exhibited a higher sensitivity (0.08 ng/mL). The inter-assay coefficient of variation (CV) and the intra-assay CV were 5.4%–7.7% and 5.7%–13.4%, respectively. The recovery rates ranged from 93% to 105%. Furthermore, a high correlation (*n* = 234, r = 0.977, *p* < 0.0001) and consistency (Kappa = 0.875) were obtained when compared with the PCT kit from Roche Elecsys BRAHMS. Thus, the new quantitative method for detecting PCT has been successfully established. The results indicated that the newly-developed system based on TRFIA combined with LFIA was suitable for rapid and on-site detection for PCT, which might be a useful platform for other biomarkers in point-of-care tests.

## 1. Introduction

Procalcitonin (PCT) is a non-hormonal precursor of the propeptide calcitonin which consists of 114 to 116 amino acids [1]. Recent studies showed that serum PCT levels are significantly elevated in bacterial infection and this has been extensively applied for diagnosis of infection [2,3]. The physiological PCT serum level is below 0.5 ng/mL, but a rise to a value higher than 2 ng/mL is indicative of sepsis [4]. Detectable levels of PCT rise in bacterial infections, but do not increase in most other inflammatory reactions, such as viral infections [5], autoimmune disease, and trauma, making PCT an excellent marker for sepsis with high specificity and sensitivity [6]. Fast and accurate detection and monitoring of PCT are very important for antibiotic treatment decision-making, especially in intensive care units (ICU) [7,8,9,10,11,12,13].

In recent years, a variety of PCT immunoassays have been available for clinical application, such as PCT detection kits based on chemiluminescence immunoassays (CLIA) [14], time-resolved fluoroimmunoassay (TRFIA), enzyme-linked fluorescent assays (ELISA) and immunochromatographic tests (ICT). Though both CLIA and TRFIA can accurately quantify the serum PCT with high sensitivity and accuracy, the large-scale testing equipment limits its use in under-equipped locales or emergency medicine wards and ICUs. On the contrary, ELISA and ICT can be quickly and easily used on site, but the accuracy is limited by their sensitivity and non-quantitative nature. Therefore, there is a pressing need to develop an accurate, rapid, quantitative and easy operating kit for detecting the special marker of PCT.

The lateral flow strip-based quantitative detection with fluorescence labeling is an emerging method and has been widely used in clinical diagnosis [15,16,17]. Compared to the conventional fluorescence labels, such as Cy-3 and Cy-5, even quantum dots, the recent reported lanthanide chelates as a fluorescence label employed to lateral flow immunoassay (LFIA) has some distinctive characteristics [18,19,20,21]. The characteristics of narrow emission spectrum, broad excitation spectrum (613 nm, 333 nm), and large Stokes shift, allows easy discrimination owing to its own unique emission signals, which eliminates the background fluorescence associated with the use of many existing fluorophores. The longer half-life of europium (Eu) (III)-chelated nanoparticles makes it ideal for use in a time-resolved fluorescence (TRF) reading system, which recognizes specific resultant signals after a certain time interval. These characteristics lead to attractive performance of a wider detection range, higher sensitivity, and accuracy. Furthermore, multi-labeling can be carried out with different lanthanide chelates for detecting biomarkers simultaneously in one test. The combination with LFIA and a portable TRF reading system offers improved detection performance for quantitative point-of-care test (POCT), such as rapidity and accuracy. This new combined system is considered a very potential one for quantitative POCT [22]. However, there is no report or product for PCT based on it. In this study, carboxylate-modified europium (III) (Eu (III)) chelate microparticles (CM-EUs) were applied to LFIA test strips for rapid quantitative and on-site detection of PCT in serum samples.

## 2. Materials and Methods

### 2.1. Materials and Reagents

PCT monoclonal antibody (MJG03) and antigen (JG01) were obtained from Hangzhou Kitgen Biotechnology Co., Ltd. (Hangzhou, Zhejiang, China). PCT monoclonal antibody (16B5) was purchased from HyTest Ltd. (Joukahaisenkatu, Turku, Finland). The monoclonal antibody MJG03 was conjugated with Eu (III) and fixed on the conjugate pad, while 16B5 was used as the capture antibody and fixed on the test line of nitrocellulose (NC) membrane. Bovine serum albumin (BSA) and the Elecsys BRAHMS PCT kit were obtained from Roche Diagnostics (Indianapolis, IN, USA). NC membrane, conjugate pad, and absorbent pad were all purchased from Millipore (Bedford, MA, USA). Goat anti-rabbit IgG was obtained from Fitzgerald Industries International, Inc. (North Acton, MA, USA). Rabbit IgG (RI-gG) was obtained from Merck and Co., Inc. (Kenilworth, NJ, USA). CM-EUs were obtained from Thermo Fisher Scientific Inc. (Reference number 93470520010150, Waltham, MA, USA). The sample pad was obtained from Jieyi Biotechnology (Shanghai, China). 1-Ethyl-3-(3-dimethylaminopropyl) carbodiimide (EDC), *N*-hydroxysulfosuccinimide (sulfo-NHS), 4-morpholineethanesulfonic acid (MES), polyvinyl alcohol (PVA), polyvinyl pyrrolidone (PVP), Trition-X100, sodium caseinate, sodium chloride, trihydroxymethyl aminomethane, and the centrifugal filter unit (with an Ultracel-50 membrane) were purchased from Sigma-Aldrich (St. Louis, MO, USA). A Milli-Q water purification system (Millipore, Bedford, MA, USA) was used for preparing ultrapure water (pH 6.5–8.5, total organic carbon 1–5 ppb). 

### 2.2. Buffer Solutions

The buffer solutions were as follows: conjugate pad treatment buffer (50 mM Na_2_HPO_4_·12H_2_O, 0.5% PVA, 1% TritonX-100, and 0.5% BSA, pH 7.4); blocking buffer (0.025 M phosphate buffer, 2% BSA, pH 7.4); binding buffer (25 mM phosphate buffer, pH 7.0); sample pad treatment buffer (0.1 M Na_2_B_4_O_7_·10H_2_O, 1% PVP, 0.2% Casein-Na, 1% TritonX-100, 1% Tetronic 1307, and 0.2% NaN_3_); washing buffer (0.025 M Tris-HCl, 0.9% NaCl, 0.2% Tween-20, and 0.05% Proclin-300, pH 7.8); activating buffer (25 mM MES, pH 6.1); sample dilution buffer (0.9% NaCl, 0.1% Proclin-300, 0.2% PEG-6000, 0.5% BSA, 0.02% Evans blue); and coating buffer (0.01 M Na_2_HPO_4_·12H_2_O, 0.9% NaCl, 0.3% Trehalose, and 0.1% NaN_3_, pH 7.4).

### 2.3. Experiment Instruments 

The BioJet Quant XYZ3060 dispenser was purchased from Biodot Ltd. (Irvine, CA, USA). The Medisensor Qcare TRF system, a portable fluorescence strip reader, was purchased from Medisensor, Inc. (Daegu, Korea) [22]. The excitation wavelength of the reader is 333 nm and the emission wavelength is 613 nm. The reader is equipped with a digital camera, an ultraviolet light source, and a front panel digital display for quantitative measurement. The probe sonicator (Scientz-IID) was obtained from Ningbo Scientz Biotechnology Co., Ltd. (Ningbo, Zhejiang, China).

### 2.4. Preparation of Standard and Controls

A series of reference standards were set at 0, 0.5, 2, 10, 20, and 40 ng/mL by diluting the PCT antigen JG01 (100 ng/mL) with the dilution buffer. Three quality controls were set at 0, 2, and 10 ng/mL by diluting JG01 with PCT-free serum.

### 2.5. Preparation of CM-EUs Coupled with Antibody

To prepare the CM-EUs-antibody conjugates (CM-EUs-Ab), 2 mg of 200 nm CM-EUs were activated for 30 min in activating buffer containing 14 µL EDC (10 mg/mL) and 132 µL sulfo-NHS (10 mg/mL) with shaking, firstly. After that, the activated CM-EUs were then washed twice with 1 mL binding buffer, and the supernatant discarded after centrifugation at 15,000× *g* for 20 min at 4 °C. Then the activated CM-EUs were resuspended in 500 μL of binding buffer by sonication. Fifty micrograms of antibody (anti-PCT MJ03 or RIgG) (purified and concentrated by a centrifugal filter unit) was added. The coupling reaction was performed for 2 h while gently blending. After removing the uncoupled antibody by centrifugation at 10,000× *g* for 20 min at 4 °C, the blocking buffer was added to the mixture, shaking for 1 h. Subsequently, the conjugate solution was resuspended by sonication. Finally, the precipitate was rinsed three times in 25 mmol/L TBS-T buffer (pH 7.2) and stored at 4 °C. 

### 2.6. Preparation of the CM-EUs Test Strip

**Figure 1:**
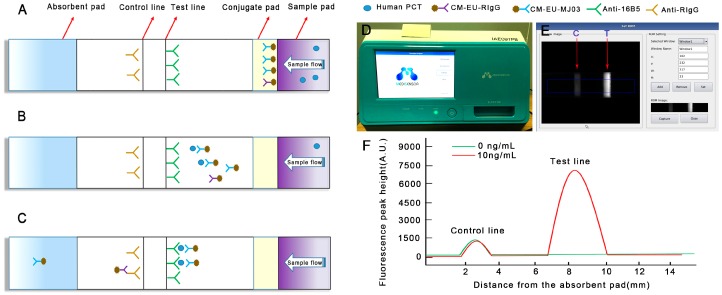
Schematic illustration of the assay procedure. (**A**) Sample containing procalcitonin (PCT) is applied to the sample pad; (**B**) PCT combines with CM-EU-MJ03 conjugates and migrates along the porous membrane by capillary action; (**C**) The formed complexes continue to migrate along the membrane and PCTs are captured by 16B5 to form CM-EU-MJ03-Ag-16B5 complexes on the test line. CM-EU-RIgG migrates continually to the control line, and is captured by anti-RIgG. The excess fluorescent microsphere continues to migrate toward the absorption pad; (**D**) The portable, small signal-acquisition device; (**E**) The preview images for “Rom setting” are selected; (**F**) The fluorescence peak height is measured by the reader.

The CM-EUs Test Strip was composed of four constituents: a sample pad, a conjugate pad, a NC membrane, and an absorbent pad (Figure 1A). Sample pad was immersed in sample pad treatment buffer for 1 h, while the conjugate pad was pretreated with conjugate pad treatment buffer for 1.5 h at room temperature, and then all were dried at 37 °C for 3 h. The CM-EUs-RIgG conjugates were diluted 100-folds in labeling antibody dilution buffer and dispensed onto a pretreated conjugate pad by XYZ3060 Dispense Workstation at the speed of 1.4 μL/mm, and then dried at 37 °C for 4 h. An optimized volume of CM-EU-MJ03 conjugate solution was dispensed onto the pretreated conjugate pad, and then dried at 37 °C for 4 h. 16B5 (2.0 mg/mL) was striped sprayed onto the test line (T) and anti-RIgG (1 mg/mL) was loaded onto the control line (C) by the XYZ3060 Dispense Workstation which had two-channel. The plate was then cut into 3 mm wide strips by a strip cutter. The prepared test strips were stored in a drying oven.

### 2.7. Serum Samples 

A total of 234 serum samples were collected from patients at Nanfang Hospital, Southern Medical University (Guangzhou, China), including 140 males and 94 females (ages from 2–97 years old). All samples were stored at −20 °C until use. The study was reviewed and approved by the clinical research ethics committee of the Southern Medical University.

### 2.8. Sample Detection and Analysis 

Initially, 30 μL of a sample (standard or serum) and 30 μL of sample dilution buffer were mixed thoroughly. The mixed solution was added onto the sample pad. Fifteen minutes later, the results of fluorescence intensity on the T line (H_T_) and the C line (H_C_) were observed by the reader. The series of reference standards (0, 0.5, 2, 10, 20, and 40 ng/mL) were set for standard curve making and signal-to-noise ratio (SNR) measuring. The fluorescence of standard 0 ng/mL (H_T0_) was set as the noise, while the fluorescence of other (H_Ti_, i refers to any of the other sample in addition to the 0 standard point) which may contain PCT as the signal. The H_Ti_/H_T0_ ratio was calculated for effectiveness evaluation. H_T0_ higher than 2000 was not accepted.

### 2.9. Statistical Analysis

The linear regression analysis, consistency analysis and Pearson’s correlation coefficient were carried out by OriginPro 7.5 (OriginLab) and SPSS 13.0 (Chicago, IL, USA). *p* < 0.05 was considered statistically significant. 

## 3. Results

### 3.1. New Detecting System Establishment and Data Judgments 

The principle of the new detecting system is shown in Figure 1. A sandwich immunoassay based on antigen-antibody reaction was employed on lateral flow test strips with a label of Eu (III). Capture antibodies (16B5 on the T line, anti-RIgG on the C line) and labeling antibodies (CM-EUs-MJ03 or CM-EUs-RIgG, on the conjugate pad) were pre-dispensed on the strips (Figure 1A). When the samples were loaded onto the sample pad, the analytes migrated to the conjugate pad and combined with CM-EUs-MJ03 (Figure 1B). After the complexes (CM-EUs-MJ03-PCT) reached the T line, they were captured by anti-PCT (16B5) and formed CM-EUs-MJ03-PCT-16B5 complexes (Figure 1C). CM-EUs-RIgG will migrate with the sample buffer alone. When they reached the C line, they were captured by anti-RIgG and formed CM-EUs-RIgG-anti-RIgG complexes (Figure 1C). After the completed reaction, the strips will be put into the portable reader device (Figure 1D). The reader, having dimensions of 348 × 240 × 221 mm, is equipped with an ultraviolet (UV) light source and a digital camera for detection, and a front display for the observation of quantitative measurement levels. The preview images for “Rom setting” will be selected at an absorption wavelength of 333 nm and an emission wavelength of 613 nm (Figure 1E). The fluorescence peak heights of the T line (H_T_) and the C line (H_C_) were measured and presented by the reader (Figure 1F). The H_T_ was used for quantitation, while H_C_ served as the internal control.

### 3.2. Optimization of Reaction Parameters

#### 3.2.1. The Optimum Amount of Capture Antibody (16B5)

**Figure 2:**
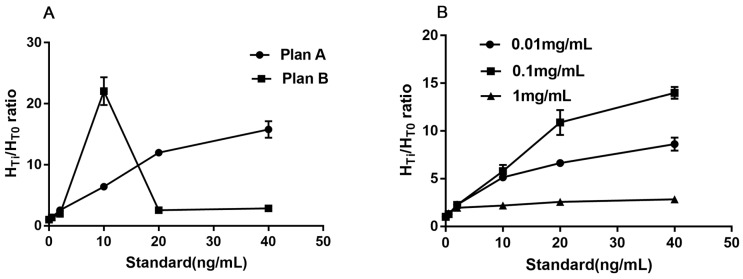
The optimized results of different sprayed speed (**A**) and concentration (**B**).

The captured antibody (16B5) was diluted to 2.0 mg/mL with coating buffer. Two different sprayed speeds were set to optimize the better quantity of 16B5. In plan A, anti-RIgG (1 mg/mL) was sprayed onto the control line (C) at a speed of 0.08 μL/mm, while the 16B5 was sprayed onto the test line (T) at a speed of 0.08 μL/mm. In plan B, anti-RIgG was handled the same as in plan A, but 16B5 was sprayed onto T line at a speed of 0.12 μL/mm. The series of reference standards (0, 0.5, 2, 10, 20, and 40 ng/mL) were used here for measuring the signal-to-noise ratio (SNR). The lower H_T0_ (higher than 2000 was not accepted), higher SNR and better linear slope are considered to have better effectiveness. The results showed that plan A has the lower H_T0_ (1138 vs. 2048), higher SNR, and better linear slope (Figure 2A). Plan A was chosen.

#### 3.2.2. The Optimum Concentration of CM-EU-MJ03 Conjugates

When the optimized sprayed speed was fixed at 0.08 μL/mm for both captured antibodies (16B5, Anti-RIgG) on the T and C line, different concentrations (0.01 mg/mL, 0.1 mg/mL, and 1 mg/mL) were set as the optimum choice of CM-EU-MJ03 conjugates at the speed of 0.08 μL/mm. The series of reference standards (0, 0.5, 2, 10, 20, and 40 ng/mL) were used here for measuring the signal-to-noise ratio (SNR). The lower H_T0_, higher SNR and better linear slope are considered to have better effectiveness. The results showed that when the concentration of CM-EU-MJ03 conjugates was at 0.1 mg/mL, it had the highest SNR and the best linear slope (Figure 2B) along with the lower H_T0_ (1277 vs. 457 for 0.01 mg/mL and 13,628 for 1 mg/mL). Thus, the concentration of 0.1 mg/mL was chosen for CM-EU-MJ03 in the future use.

### 3.3. Performance Evaluation

Based on the above optimization experiments, we prepared small batch products with the optimized parameters. All of the test strips were dried at room temperature with relative humidity below 40% for 4 h, cut (3 mm width), and placed in disposable cassettes. All of the products were stored in a drying oven for further evaluation.

#### 3.3.1. Analytical Sensitivity and Linearity

**Figure 3:**
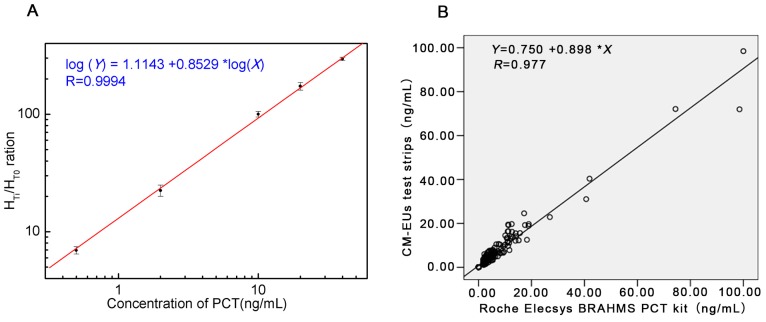
The standard curve and the results of correlation analysis. (**A**) Standard curve for PCT; (**B**) Comparison of PCT in 234 sera samples measured with the developed CM-EUs test strips and a Roche Elecsys BRAHMS PCT kit.

A standard curve was obtained based on the measurement of six serial conference standards (0, 0.5, 2, 10, 20, and 40 ng/mL). The fluorescence of standard 0 ng/mL (H_T0_) was set as the noise, while the fluorescence of other standards (H_Ti_, i refers to any of the standard point besides 0) which contains the PCT antigen (JG01) as the signal. The H_Ti_/H_T0_ ratio was calculated. The standard curve was carried out by plotting the logarithm of H_Ti_/H_T0_ (y) against the logarithm of the PCT concentration (x). Under those optimized conditions, we obtained a reasonable calibration curve for the proposed assay (Figure 3A). The equation of the regression curve was log(y) = 1.1143 + 0.8529log(x), Pearson’s correlation coefficient (r = 0.9994). The analytical sensitivity of the proposed assay was 0.08 ng/mL, defined as the mean plus two SD (*n* = 20) of the zero standard. These results showed the excellent performance of the developed CM-EU test strips.

#### 3.3.2. Accuracy

The accuracy of the assay was evaluated by a recovery test. Measuring with a serum sample with a high concentration of PCT (62.8 ng/mL, measured by Roche Elecsys BRAHMS PCT kit) was diluted into three dilutions. The recovery ratios (measured value/expected value) were calculated. The results showed that they were between 0.93 and 1.05 (Table 1).

#### 3.3.3. Precision

Duplicate tests were performed with three reference standard concentrations set as low (standard I: 0.5 ng/mL), moderate (standard II: 2.0 ng/mL), and high (standard III: 10 ng/mL). The inter-assay CV ranged from 5.4% to 7.7% and the intra-assay CV ranged from 5.7% to 13.4% (Table 2).

#### 3.3.4. Specificity

The CRP (50 ng/mL), IL-6(1 IU/mL), human calcitonin (60 ng/mL), and human anti-calcium (30 ng/mL) were measured. All of the results were less than 0.08 ng/mL (Table 3). This indicated that the developed CM-EUs test strips had no cross-reaction with CRP, IL-6, human calcitonin, and anti-human calcium.

### 3.4. Clinical Samples Analysis

A total of 234 patients’ serum samples were measured with the developed CM-EUs test strips and Roche Elecsys BRAHMS PCT kit. The results of correlation analysis were presented in Figure 3B. A high correlation was obtained between the two assays (y = 0.898x + 0.750, r = 0.977, *p* < 0.0001). Furthermore, when samples were divided into four groups according the determination levels of the control kit (<0.5 ng/mL, 0.50~2.00 ng/mL, 2.00~10.00 ng/mL, and >10.00 ng/mL) the Kappa value was 0.875 (Table 4). Therefore, the developed LFIA combined with TRFIA system for PCT showed excellent performance compared to Roche Elecsys BRAHMS PCT kit. 

## 4. Discussion

In recent decades, PCT has been regarded as a useful marker in the clinic. Serum PCT levels will rise significantly above normal in patients with sepsis and other bacterial infections. The induction period of PCT (4 to 12 h) is longer than cytokines, but shorter than C-reaction protein (CRP) [23]. PCT is also a relatively stable protein with a half-life of about 22 to 35 h [24]. PCT reflects the effectiveness of antibiotics and may be used to guide antibiotic treatment, reduce the abuse of antibiotics which may benefit the reduction of the waste of treatment and avoid bacterial drug resistance [7,8]. A rapid, user-friendly, inexpensive, and quantitative method for PCT detection is very necessary to meet the widespread application in the clinic.

The newly-developed system was a double-antibody sandwich immunofluorescent assay based on TRFIA combined with LFIA. The test can be finished within 15 min and provides quantitative results on-site by a portable reader device. The comparative study in 234 clinical samples showed that the newly-developed system had excellent consistency with a Roch Elecsys BRAHMS PCT (r = 0.977, Kappa = 0.875), but it takes 25 min for the Roch Elecsys BRAHMS PCT, which is a much larger analytic instrument, limiting the availability in under-equipped locales. The colloidal gold kit can be quickly and easily used o-site, but cannot accurately quantify. 

Since the non-uniformity between the properties of lateral flow strips is the limitation for quantitative detection with lateral flow strips, several strategies have been undertaken to achieve ideal repeatability and accuracy in our work. First, we selected a desirable nitrocellulose (NC) membrane which may best fit the design requirement through the specification provided by manufacturers and the experience from our laboratory team [25,26,27]. Different membranes have different physical and chemical attributes, which affect its capillary flow properties. The capillary flow properties, in turn, affect reagent deposition, assay sensitivity, assay specificity, and test line consistency. When the capillary flow rate is relatively faster, it will finish in a shorter time, and will likely consume more reagents and have reduced sensitivity. The magnitude of the effects and the limits of acceptability are unique to the assay, reagents, and manufacturer. To select a desirable membrane, the balances between speed, sensitivity, and cost should be taken into consideration. Assays consuming less reagents and achieving higher sensitivity may be the most desirable for infectious disease testing. In our study, PCT is a marker for diagnosis and differential diagnosis of bacterial infection from virus infection. According to the specification provided by manufacturers and the experience from our laboratory team, HF135 was selected, which may best fit the design requirement of rapidity and sensitivity. A confirmatory experiment was also conducted. The results showed that the selected membrane (HF135) had the higher fluorescence value for positive results, a lower fluorescence signal for the negative background, and good homogeneity (Data not shown). Second, the concentration and the sprayed speed of the samples online were optimized to avoid sample stacking. Third, an automatic two-channel spray device was enrolled to achieve the spray precision and uniformity. Fourth, the technological process and quality control are critically demanded during the pads, and membranes overlapped one another to guarantee the consistency that the flow dynamics are uniform on all of the strips manufactured. Last, we calculate the H_Ti_/H_T0_ ratio (SNR) to evaluate the effectiveness to counteract the interference and non-uniformity caused by the sample matrix and strips. The repeatability results showed that the inter-assay CV were no more than 7.7% and the intra-assay CV ranged from 5.7% to 13.4%. The accuracy results showed that the recovery ratios were between 0.93 and 1.05. They are all highly acceptable for detection performance.

## 5. Conclusions

A rapid, sensitive, quantitative kit for on-site detection of PCT in human serum has been developed. Compared with the existing similar products, the highlights of the research work are as follows: (1) This method employs the lanthanide chelated nanoparticles (CM-EUs) into the method based on LFIA as a fluorescence label for PCT detection, which has distinctive characteristics to guarantee high sensitivity and accuracy. The combination with LFIA and TRFIA offers improved detection performance for quantitative POCT; (2) To counteract the interference and non-uniformity caused by the sample matrix and strips, the H_Ti_/H_T0_ ratio (SNR) was calculated for effectiveness evaluation; and (3) Quantitative detection by using a conference standard curve along with H_Ti_/H_T0_ ratio model has been achieved. All of the results and the advantages indicated that the new developed system based on TRFIA combined with LFIA might be a useful platform for other biomarkers in quantitative point-of-care tests.

## Figures and Tables

**Table 1:** Accuracy results of the CM-EU test strips (*n* = 3).

Sample	1:2	1:4	1:8
Expectation (ng/mL)	31.40	15.70	7.85
Mean value ± SD (ng/mL)	29.96 ± 2.05	16.85 ± 1.05	7.94 ± 0.55
Recovery	1.05	0.93	0.99

**Table 2:** Precision results of the CM-EU test strips.

References (ng/mL)	Inter-Assay Precision (*n* = 5)		CV ^2^ %	Intra-Assay Precision (*n* = 3 × 2)		CV %
	Mean (ng/mL)	SD ^1^ (ng/mL)		Mean (ng/mL)	SD (ng/mL)	
0.5	0.502	0.032	6.4	0.53	0.036	6.8
2.0	2.08	0.16	7.7	2.17	0.29	13.4
10.	10.16	0.55	5.4	10.35	0.59	5.7

^1^ SD: Standard Deviation; ^2^ CV: Coefficient of Variation.

**Table 3:** Precision results of the CM-EU test strips.

Concentration of the Sample	The Specificity of the Sample	Test Results (ng/mL)
50 ng/mL	CRP	<0.08
1 IU/mL	IL-6	<0.08
60 ng/mL	Human calcitonin	<0.08
30 ng/mL	Human anti-calcium	<0.08

**Table 4:** Consistency analysis of the two assays.

CM-EU-Based Tests Trip Assay (ng/mL)	Roche Elecsys BRAHMS PCT Kit (ng/mL)				Total
	<0.50	0.05 ≤ PCT ≤ 2.00	2.00 < PCT ≤ 10.00	>10	
<0.50	83	0	0	0	83
0.05 ≤ PCT ≤ 2.00	0	5	8	0	13
2.00 < PCT ≤ 10.00	0	6	95	2	103
>10.00	0	0	3	32	35
Total	0	11	106	34	234

## References

[1] Lipinska-Gediga M., Mierzchala-Pasierb M., Durek G. (2016). Procalcitonin kinetics—Prognostic and diagnostic significance in septic patients. Arch. Med. Sci..

[2] Du B., Pan J., Chen D., Li Y. (2003). Serum procalcitonin and interleukin-6 levels may help to differentiate systemic inflammatory response of infectious and non-infectious origin. Chin. Med. J..

[3] Uzzan B., Cohen R., Nicolas P., Cucherat M., Perret G.Y. (2006). Procalcitonin as a diagnostic test for sepsis in critically ill adults and after surgery or trauma: A systematic review and meta-analysis. Crit. Care Med..

[4] Becker K.L., Snider R., Nylen E.S. (2008). Procalcitonin assay in systemic inflammation, infection, and sepsis: Clinical utility and limitations. Crit. Care Med..

[5] Riedel S., Melendez J.H., An A.T., Rosenbaum J.E., Zenilman J.M. (2011). Procalcitonin as a marker for the detection of bacteremia and sepsis in the emergency department. Am. J. Clin. Pathol..

[6] Reinhart K., Meisner M., Brunkhorst F.M. (2006). Markers for sepsis diagnosis: What is useful?. Crit. Care Clin..

[7] Hohn A., Schroeder S., Gehrt A., Bernhardt K., Bein B., Wegscheider K., Hochreiter M. (2013). Procalcitonin-guided algorithm to reduce length of antibiotic therapy in patients with severe sepsis and septic shock. BMC Infect. Dis..

[8] Bouadma L., Luyt C.E., Tubach F., Cracco C., Alvarez A., Schwebel C., Schortgen F., Lasocki S., Veber B., Dehoux M. (2010). Use of procalcitonin to reduce patients’ exposure to antibiotics in intensive care units (PRORATA trial): A multicentre randomised controlled trial. Lancet.

[9] Christ-Crain M., Muller B. (2005). Procalcitonin in bacterial infections—Hype, hope, more or less?. Swiss Med. Wkly..

[10] Charles P.E., Ladoire S., Aho S., Quenot J.P., Doise J.M., Prin S., Olsson N.O., Blettery B. (2008). Serum procalcitonin elevation in critically ill patients at the onset of bacteremia caused by either Gram negative or Gram positive bacteria. BMC Infect. Dis..

[11] Novotny A., Emmanuel K., Matevossian E., Kriner M., Ulm K., Bartels H., Holzmann B., Weighardt H., Siewert J.R. (2007). Use of procalcitonin for early prediction of lethal outcome of postoperative sepsis. Am. J. Surg..

[12] Jones A.E., Fiechtl J.F., Brown M.D., Ballew J.J., Kline J.A. (2007). Procalcitonin test in the diagnosis of bacteremia: A meta-analysis. Ann. Emerg. Med..

[13] Giamarellos-Bourboulis E.J., Giannopoulou P., Grecka P., Voros D., Mandragos K., Giamarellou H. (2004). Should procalcitonin be introduced in the diagnostic criteria for the systemic inflammatory response syndrome and sepsis?. J. Crit. Care.

[14] Fu Z., Yan F., Liu H., Lin J., Ju H. (2008). A channel-resolved approach coupled with magnet-captured technique for multianalyte chemiluminescent immunoassay. Biosens. Bioelectron..

[15] Wang L., Lu D., Wang J., Du D., Zou Z., Wang H., Smith J.N., Timchalk C., Liu F., Lin Y. (2011). A novel immunochromatographic electrochemical biosensor for highly sensitive and selective detection of trichloropyridinol, a biomarker of exposure to chlorpyrifos. Biosens. Bioelectron..

[16] Zhou Y., Zhang Y., Pan F., Li Y., Lu S., Ren H., Shen Q., Li Z., Zhang J., Chen Q. (2010). A competitive immunochromatographic assay based on a novel probe for the detection of mercury (II) ions in water samples. Biosens. Bioelectron..

[17] Hua X., Qian G., Yang J., Hu B., Fan J., Qin N., Li G., Wang Y., Liu F. (2010). Development of an immunochromatographic assay for the rapid detection of chlorpyrifos-methyl in water samples. Biosens. Bioelectron..

[18] Zhang F., Zou M., Chen Y., Li J., Wang Y., Qi X., Xue Q. (2014). Lanthanide-labeled immunochromatographic strips for the rapid detection of Pantoea stewartii subsp. stewartii. Biosens. Bioelectron..

[19] Xu W., Chen X., Huang X., Yang W., Liu C., Lai W., Xu H., Xiong Y. (2013). Ru(phen)3(2+) doped silica nanoparticle based immunochromatographic strip for rapid quantitative detection of beta-agonist residues in swine urine. Talanta.

[20] Xia X., Xu Y., Ke R., Zhang H., Zou M., Yang W., Li Q. (2013). A highly sensitive europium nanoparticle-based lateral flow immunoassay for detection of chloramphenicol residue. Anal. Bioanal. Chem..

[21] Xia X., Xu Y., Zhao X., Li Q. (2009). Lateral flow immunoassay using europium chelate-loaded silica nanoparticles as labels. Clin. Chem..

[22] Ham J.Y., Jung J., Hwang B.G., Kim W.J., Kim Y.S., Kim E.J., Cho M.Y., Hwang M.S., Won D.I., Suh J.S. (2015). Highly sensitive and novel point-of-care system, aQcare Chlamydia TRF kit for detecting Chlamydia trachomatis by using europium (Eu) (III) chelated nanoparticles. Ann. Lab. Med..

[23] Meisner M. (2002). Pathobiochemistry and clinical use of procalcitonin. Clin. Chim. Acta.

[24] Reinhart K., Karzai W., Meisner M. (2000). Procalcitonin as a marker of the systemic inflammatory response to infection. Intensive Care Med..

[25] Liang R.L., Xu X.P., Liu T.C., Zhou J.W., Wang X.G., Ren Z.Q., Hao F., Wu Y.S. (2015). Rapid and sensitive lateral flow immunoassay method for determining alpha fetoprotein in serum using europium (III) chelate microparticles-based lateral flow test strips. Anal. Chim. Acta.

[26] Lai X.H., Liang R.L., Liu T.C., Dong Z.N., Wu Y.S., Li L.H. (2016). A Fluorescence Immunochromatographic Assay Using Europium (III) Chelate Microparticles for Rapid, Quantitative and Sensitive Detection of Creatine Kinase MB. J. Fluoresc..

[27] Wang X., Zhang Q., Hao F., Gao X., Wu W., Liang M., Liao Z., Luo S., Xu W., Li D. (2016). Development of a colloidal gold kit for the diagnosis of severe fever with thrombocytopenia syndrome virus infection. Biomed. Res. Int..

